# Qualitative differences in the mindsets associated with dual nature of normative commitment

**DOI:** 10.1371/journal.pone.0251193

**Published:** 2021-06-17

**Authors:** Hyun Sung Oh, Sukanlaya Sawang

**Affiliations:** 1 Jeonju University, Jeonju, South Korea; 2 Coventry University, Coventry, United Kingdom; sapienza, ITALY

## Abstract

This study aims to o uncover how employees’ normative commitment (sense of obligation) to their organization is experienced in terms of dual normative commitment (moral imperative or indebted obligation) and to describe the potential for different mindsets arising through the dynamic combination of the various components in the commitment profile. Semi-structured interviews were conducted with 16 participants. The interviews were designed to identify the respondents’ perceptions of obligation to their organisation, and their underlying motivational mindset associating with dual nature of normative commitment The interview findings for the affective-normative commitment dominant and the continuance commitment dominant participants were consistent with normative commitment experienced as either moral imperative or an indebted obligation, depending on the relative levels of affective and continuance commitment. All participants irrespective of their commitment profile noted that they had commitment to multiple foci, however, the alignment between commitment to these various foci differed by commitment profile. The qualitative differences among the commitment profiles indicated that the interaction of the commitment components is more complex than current commitment profile propositions suggest and that further theory development beyond the mindsets associated with continuance commitment and affective-normative commitment dominant profiles is required.

## Introduction

Organisational commitment is extensively represented in the human resource management and organisational behaviour literature as a key factor in the relationship between employees and their organisations. Although Allen and Meyer [1] noted that an employee can experience the three components of organisational commitment simultaneously, in terms of commitment profiles, the majority of studies have examined the antecedents and outcomes of affective, continuance, and normative commitment, independently. The various combinations of the three components are proposed to generate qualitatively different mindsets that have important implications for employee work-related behaviours [2–4].

The current study aims to qualitatively explore the relationship between mindsets and Organizational Commitment (OC). The contributions are twofold. First, the qualitative study of mindsets and OC is limited and future study should employ the qualitative research analysis to deepen our understanding of OC [5, 6]. Meyer and associates [2–4] theorised that there were rich insights to be gained into employees’ different mindsets associated with various commitment profiles. Despite an expanding body of research on commitment profiles, the existence of qualitative differences in the mindsets associated with the profiles has only been inferred from the patterns of results from survey studies [7]. In the context of OC, Meyer and Herscovitch [8] described mindset as a bond between an employee and an organisation that engenders employees’ commitment to action that is consistent with the stated goals of an organisation. The current study focuses on mindset as it pertains to the established set of attitudes held by an employee towards his or her current organisation and his or her role, relationships, and situational circumstances within the organisation.

Second, the current research provides new insights into the role of normative commitment when interacts with both affective and continuance commitments. A growing body of research uses a profile approach, generating a commitment profile for each employee that represents a configuration of the strengths of the three OC components [8]. An employee’s commitment profile consists not only in the distinct levels of the three components of his or her OC (i.e., affective, normative, and continuance commitments) but in the interaction among these components. An important conceptual paper by Meyer and colleagues [2], which elaborates on earlier work by Gellatly et al. [9], introduces the possibility of normative commitment (employees’ sense of obligation to the organisation) having a “dual nature”. It is concluded that normative commitment may have a dual nature depending upon the context in which it is experienced, that is, the two cases mentioned are: a) high affective with low continuance commitment, and b) low affective with high continuance commitment. The current study further explores this dual concept of normative commitment by exploring how the dual nature of normative commitment produces an apparent “mindset” in employees that in turn influences an employee’s work-related behaviour.

### Context effect on commitment profile

A context effect, as conceived by Gellatly et al. [9], refers to the potential for a particular component within the commitment profile to be influenced by the strengths of the other components present. In particular, Gellatly et al. [9] proposed that the nature of normative commitment might be experienced as either moral imperative or indebted obligation, depending on the context provided by the relative strengths of affective and continuance commitment within an employee’s commitment profile. This conceptual framework reflects the dual nature of normative commitment. It is proposed that, when affective is dominant, normative commitment will be experienced as a moral imperative. On the other hand, when continuance commitment is dominant, normative commitment will be experienced as an indebted obligation.

However, this proposition has only been inferred from differences observed in the relationship between commitment profiles and outcome variables. The assertion that different commitment profiles produce distinct mindsets which generate different cognitive and affective reactions remains untested. Meyer and colleagues [2] noted that “the unique combinations of the affective, continuance and normative commitments appear to produce *qualitatively* different mindsets that have important implications for behaviour” (p. 287; emphasis added). The current study thus aims to address the need for qualitative research on the mindsets associated with commitment profiles and the influence of context effects on the nature of normative commitment.

Expanding on the conceptual work of normative commitment—moral imperative or indebted obligation—[9], Meyer and colleagues [2] explained how such a dual nature of normative commitment, associated with mindsets, might develop in accordance with the motivational mechanisms that underpin employees’ commitment profiles. Key motivational mechanisms that influence employees’ commitment include self-determination theory (SDT), perceived organisational support (POS), psychological contract (PC), and experience of leadership (EL). The following section reviews Meyer and colleagues’ [2] propositions and the theoretical frameworks from which they are drawn (SDT, POS, PC and EL) in order to clarify the link between commitment profile and mindset. Moreover, these four theories will be used as a broad framework to the discussion of results. This section will not discuss each theory in detail but instead will provide the discussion around how these theories act as motivation mechanism for individuals to develop associative mindset with commitment profiles.

Table 1 provides a summary of the differences between a moral imperative and an indebted obligation with respect to the motivational mechanisms which have been reviewed.

**Table 1:** Commitment profiles formation based on dual nature of normative commitment.

	Moral Imperative	Indebted Obligation
Commitment Profile	High affective and normative and low continuance commitments.	High continuance and normative and low affective commitments.
Mindset	Strong desire to pursue a course of action because it is the right and moral thing to do.	A sense of having to pursue an action to avoid the social costs for failing to do so.
	Strong need to reciprocate with a broad view of what is included in the terms of commitment.	Restrict their obligations to the organization to the explicit terms of their employment contract.
Belief	Positive beliefs about organization (inherent goodness, and meaningfulness).	Less positive beliefs (e.g., indebtedness, inconvenience).
SDT	Greater levels of autonomous forms of regulation (i.e., intrinsic and integrated).	More controlled forms of regulation (i.e., external and introjected)
POS	High level of POS.	Lower levels of POS.
	Value congruence with organizational values.	Lower levels of shared values.
PC	Ideological infused psychological contract or	Transactional psychological contract.
	Relational psychological contract.	
EL	Transformational, charismatic and authentic leadership.	Transactional leadership without accompanying transactional leadership behaviors.

Note: SDT = self-determination theory; POS = perceived organizational support; PC = psychological contract; EL = experience of leadership.

#### Self-determination theory (SDT)

Meyer et al. [10] integrated commitment and motivation theory by highlighting the similarities between the mindsets associated with the components of commitment and the motivational states identified by Deci and Ryan [11]) and Ryan and Deci [12] in SDT. They noted that both commitment and motivation can be considered as an “energising force with implications for behaviour” in terms of internal drive [10]. Meyer et al. [10] argued that employees practise their motivational state in diverse ways and employees’ job outcomes can be different depending on autonomous forms of regulation. For example, employees’ motivational states can be associated with attaining rewards, avoiding punishment and shame or achieving values and self-expression. Thus, employees’ job outcomes can be differently derived from autonomous forms of regulations.

It is proposed that when combined with strong affective commitment, normative commitment is associated with autonomous regulation. Employees who experience obligation as a moral duty will fully support organisational goals (because they are value congruent) and devote effort to attaining these goals even under difficult conditions. On the other hand, introjected motivation underlies indebted obligation within normative commitment. When this is found in combination with strong continuance commitment, it manifests itself as an indebted obligation. For example, employees who are motivated to meet the expectations of others (introjected regulation) are less productive and their attitude is less healthy because they operate on lower levels of discretionary effort [2].

#### Perceived Organisational Support (POS)

Research on POS has found that not only does POS lead to the development of affective commitment but it also generates a felt obligation to the organisation [13–15]. POS leads to a heightened desire to reciprocate to the organisation with increased effort [16]. When normative commitment is combined with a strong affective commitment, the nature of employees’ obligation can be experienced as a moral imperative because he or she believes that the treatment being received from the organisation is largely positive in terms of favourable working conditions, organisational rewards, fair treatment, and support from supervisors. In such a case, employees’ positive emotional attachment to their organisation increases. Consequently, this positive emotional desire leads to employees’ moral imperative obligation because the employees feel a heightened desire to reciprocate with increased effort to help the organisation achieve its objectives in terms of the norm of reciprocity. This aligns with the previous research on POS. When the organisation provides their employees with fair organisational support, POS leads to an employee’s positive beliefs toward the organisation [17, 18].

#### Psychological Contract (PC)

PC can be classified as transactional and relational contracts. Transactional contracts are associated with specific, short-term and financial obligations (e.g., pay for service) that require a limited contribution from each party. In contrast, relational psychological contracts are associated with an open-ended time frame and long-term obligations, and are founded on the exchange of socio-emotional elements such as support and loyalty, not only monetisable elements (e.g., pay for service) [19].

In addition to both relational and transactional psychological contracts, there is a new form of psychological contract called the ideology-infused psychological contract. An ideology-infused contract is based on a shared obligation to advance a cause or ideology valued by both employer and employee [20]. It is likely to develop when employees’ values align with those of the organisation, giving them a shared meaning or purpose. As a result, employees are more likely to put effort into their work and to cooperate with their organisation as they believe that this is the right thing to do for the cause. Thus, Meyer and colleagues [2] proposed that the nature of an employee’ mindset is associated with an ideology-infused contract. The authors state that both relational psychological and ideology-infused contracts are more likely to develop an affective / normative commitment-dominant profile. They do not differentiate between the natures of normative commitment associated with these two types of contracts.

#### Experience of Leadership (EL)

The transformational leader is more likely to produce a moral imperative in employees because transformational leaders are more likely to exert transformational moral influence over followers in the long-term and display ethical leadership based on their morality [2]. Consequently, it has been argued that employees with leaders, who use transformational leadership, are more likely to stay with their organization and are willing to put effort into their work, because their attitudes toward the organization are driven by their positive relationship with its leaders. In addition to employee OC, it can be expected that employees will commit themselves to different foci; for example, a supervisor, their occupation, or a career [21]. On the other hand, employees with a transactional leader are more likely to manifest indebted obligation, as the transactional leader is more exclusively interested in achieving his or her own perceived objectives, thus fostering a sense of controlled motivation [2].

In sum, normative commitment is more likely to be experienced as a moral imperative when an employee is affectively attached to the organization, or where a relational or an ideology-infused psychological contract applies: for instance, if employees experience value congruence with the organization, a long-term, high-quality relationship with the principal supervisor, or more autonomous forms of regulation [2]. Thus, the nature of an employee’s moral imperative is driven by his or her positive beliefs about the organization, where he or she perceives a positive long-term relationship with the organization and believes that the organization cares for and treats them well. On the other hand, the mindset associated with indebted obligation is precipitated by the perception of a short-term relationship with the organization based on a transactional psychological contract, more controlled forms of regulation, and transactional leadership.

#### Current study

In summary, the concept of two faces of normative commitment requires further research, such as an investigation of the most prevalent factors at play in employees’ experience. This study aims to uncover how employees’ normative commitment (sense of obligation) to their organization is experienced in terms of dual normative commitment (moral imperative or indebted obligation) and to describe the potential for different mindsets arising through the dynamic combination of the various components in the commitment profile.

## Materials and methods

### Respondents

Sixteen respondents were recruited from Master of Business Administration (MBA) class at a large Australian University. According to Guest et al. [22] and others [23, 24], 12–16 interviews are the adequate sample size to achieve thematic saturation in a qualitative study. The current study could retrieve salient themes and reached thematic saturation within 16 interviews. This study was approved by Queensland University of Technology’s Human Research Ethics Committee. Before proceeding with interviews, all interviewees were informed that their personal information would be treated confidentially and that the study was conducted in accordance with the ethical standards. *All interviewees were asked to sign an “informed consent form” and their consent to audio-record the interview was also sought and gained.

The sample of this study was the MBA students who were currently working full-time and therefore were able to provide the reflection in term of organisational commitment. There were 6 female and 10 were male. The age range of the participants was 25 to 64 (mean = 43 years), with an average employment tenure in their current position of 9.5 years. The demographic summary of the sample is presented in Table 2.

**Table 2:** Demographics of interview respondents.

ID	Age	Occupation	Duration of work	Commitment Profiles*
Respondent 1	40	Senior research consultant	9 years 9 months	Uncommitted
Respondent 2	34	CEO	3 years 4 months	Uncommitted
Respondent 3	37	School Teacher	12 years 2 months	Moderate affective commitment
Respondent 4	64	Academic	7 years	High continuous commitment
Respondent 5	25	System Engineer	02 years 1 months	Moderate continuance -normative commitments
Respondent 6	42	Public officer	22 years 9 months	Moderate continuance -normative commitments
Respondent 7	32	Finance officer	7 years 8 months	Moderate affective commitment
Respondent 8	35	State Finance Manager	11 years	Moderate affective commitment
Respondent 9	55	Sessional Academic	5 years	Uncommitted
Respondent 10	34	High School Teacher	12 years	High affective-normative commitment
Respondent 11	37	Financial analyst	5 years 6 months	High affective-normative commitment
Respondent 12	51	Commercial Manager (CFO1)	1 year	Moderate continuance -normative commitments
Respondent 13	50	Mechanical Engineer	26 years	Moderate affective commitment
Respondent 14	55	Commercial Manager (CFO2)	14 years	High affective-normative commitment
Respondent 15	55	CHR	7 years 8 months	Uncommitted
Respondent 16	55	L & D Manager	1 year	Moderate affective commitment

Note

*Respondents were as to complete the commitment profile survey prior the interview. The survey was conducted among 108 MBA students, a cluster analysis was undertaken to identify commitment profile. The Cronbach alphas were higher than .7 for all questions.

### Interview study

This study was approved by Queensland University of Technology’s Human Research Ethics Committee. The interviews were semi-structured, carried out by the first author and lasted between 50–60 minutes. Each interview was tape recorded and supplemented by written notes or memos made by the interviewer. A semi-structured interview protocol was developed to enable standardisation of the data collection ensuring that all interviewees were guided towards discussing the same topic areas. Each interview consisted of five sections. First, participants were asked to describe their role and what aspects of their work experience they liked and disliked. Second, they were asked about their employment relationships and the expectations they have of their organization, the expectations the organization had of them, and the extent to which these expectations have been met, and what the consequences of this were. Third, participants were asked to identify, and comment on, what obligations they perceived they had to their organization. Fourth, participants were asked to describe the leadership style of their supervisors and to comment on how their supervisor’s leadership played a role in shaping their perception of the organization (see S1 Appendix for the interview protocol). All interviews were last approximately one hour and were audio-recorded and transcribed for analysis. A multi-step thematic analysis approach was utilised, i.e. the coding of text; the development of descriptive themes; and the generation of analytical themes—to answer research questions.

## Results

Participants made a total of 146 distinct comments regarding obligations. These comments were classified into seven themes. These potential themes were then communicated to an independent rater. Classification agreement between the independent rater and the researcher was high at 85.61% (κ = .84, t = 35.03, p < .05). The seven themes are presented also in Table 3.

**Table 3:** Results of the thematic analysis of features of employee perceptions of obligation.

Themes	No. of participants with comment(s) on theme	Total no. of comments*	Sample comment
*Nature of Obligation*			
Moral imperative/duty	11	27	*The owners have shown so much faith in my ability and provided me with the opportunity of expanding my knowledge of the business*, *I guess I feel that I’m obliged to provide them good returns for that investment*, *I want to deliver them great outcomes*.
Indebted obligation	10	20	*It is purely economic necessity*, *it is extremely difficult to find another job*, *so work for me is just a contractual thing–they pay me and I work for them*.
Absence of obligation	4	12	*I feel no obligation to stay; no obligation to work extremely hard; no obligation to do*, *you know*, *my utmost*. *Just because of the way that we’d been treated*.
*Focus of Obligation*			
Organization	13	27	*I feel very loyal to my school*, *I do feel obligated to do what I would think is the right thing by them*, *to do the job to a particular standard and all that entails*.
Supervisor	7	23	*To be offered the state accountant role*, *that was unheard of (for a female) so it was due to the faith of the general manager at the time*, *I owe him the world*.
Co-workers/Subordinates	6	19	*The only obligations I have are to the people who work for me and those that I work with in my department–I don’t feel obliged to the larger organization*.
Students/Clients/Patients	10	18	*I do not see my obligations to my employer but I am more inclined to see them to the students*.

Note

*number of comments can exceed number of participants, as a participant may have made multiple comments on the same theme.

The themes are divided into two types: nature of obligations and obligations to specific foci. Nature of obligations were categorised as either moral imperative, indebted obligation, or absence of obligations. The moral imperative category reflects (I) high affective and high normative commitment and (II) moderate affective and moderate continuance commitment. The moral imperative mindset results in an individual striving to complete his or her tasks as it is the right thing to do and he or she feels willing to do it [9]. Employees with this mindset are also expected to exert additional discretionary effort to achieve organisational objectives, even if it is not specified in the terms of their employment contract [9]. The indebted obligation category reflects (I) high continuance and (II) moderate continuance and moderate normative commitment, arising from a sense of having to pursue an action to avoid the social costs of failing to do so, and a restriction of felt obligations to the explicit terms of an employment contract. An indebted obligation is also associated with perceived cost, with the terms of the obligation based on a sense of having to do something to avoid the social cost of not doing so. The absence of obligation category consisted of statements where participants experienced or perceived no obligation to the organisation.

The second category of themes concerned obligations to specific foci; these were the organization, supervisor, co-workers and subordinates, and customers / students / patients / clients. The discussion of results is structured by nature of obligation (moral imperative, indebted obligation, or absence of obligations) as follow.

### Moral imperative I: High affective and normative

The high affective and high normative profile individual was expected to have a mindset which resulted in obligations characterised as moral imperatives [2, 9]. Respondents provided responses that were consistent with a mindset fostering moral imperative obligations, stressing a desire to reciprocate with their organization because it is the “right thing to do”. Felt obligations, incorporating organizational reciprocity, went hand-in-hand with statements of positive beliefs about the organization and feelings that the organization and its agents valued their contribution and cared about their well-being. This is consistent with POS research that has found that not only does POS lead to the development of affective commitment but that it also generates a felt obligation to the organization [15]. All participants within this profile noted a strong need to reciprocate the positive support they had received from their organization.

“*It’s a quid pro quo thing–you know–they’ve looked after me and now I’m going to ensure I look after them*. *I’ve made a commitment to this organization and I want to demonstrate to them that the investment they have made in me is a worthwhile and of general benefit to the firm*.*” (Participant 11 –financial analyst)*

In conjunction with strong perceptions of POS were participants’ perceptions that their organizational leadership and direct supervisors valued and respected them. This is also consistent with POS research which has repeatedly found that supervisor support and quality is strongly linked to POS [17, 25]. An important element of this relationship was mutual trust through the provision of autonomy in how work would be carried out. This pattern of findings is also consistent with Meyer and Parfyonova’s [2] proposition that employees within this profile will be more likely to experience autonomous forms of regulation and transformational leadership.

“*I feel very loyal to my school*, *I do feel obligated to my school particularly because my leadership team have (sic) shown me a lot of flexibility over the years na they’ve given me that flexibility*, *and trusted me not to abuse it*.*” (Participant 10 –high school teacher)*

All participants within this profile had a relational psychological contract is consistent with recent research findings [26] and theorising [2] on the association between OC and psychological contracts. The relationship between normative commitment and ideology-infused psychological contract was also evident. Three of the four high affective-normative employees noted the importance of value alignment as reflected in the following comment:

“*I like working with high school kids because of the level of interaction that you can have with them*, *and the conversations you can have with them*. *You can have a conversation that has some sort of intellectual capacity*. *And you can really*…*the other thing I like you can feel like you might actually make a difference in their learning and education and make them better students*. *I like my current school as I feel it enables me to be a good teacher*.*” (Participant 10 –high school teacher)*

Interestingly, the one high affective-normative employee who did have not an ideology-infused psychological contract explicitly noted that the company he was working for was the best he had ever worked for, but he noted that it was unfortunate that it did not align with his own personal passion. He clearly had a relational psychological contract with the organization but not an ideology-infused one. He further noted that he is staying with his current organization more out of necessity than personal obligation to the organization.

“*I think you always see*, *as a company… Looks after their staff; they are one of those few companies… Well it’s the only company I’ve ever worked for that genuinely*, *what they do matches the rhetoric I suppose*. *Or I suppose it’s not rhetoric because they mean it*. *They’re one of the few*, *especially in financial services that I think value staff*, *and try and keep you happy genuinely*. *I just think I’m one of the few people that really enjoy the place that they work for and recognise how good of an employer they are*. *But saying that*, *it’s just a shame that it doesn’t align with my passion*, *if financial services were my passion*, *and I was working for an employer like XXX*, *it would be a double win*. *But unfortunately it’s not the case*.*”(Participant11 –financial analyst)*

### Moral imperative II: Moderate affective and continuance

Participants within this profile described their relationship in terms of a relational psychological contract. The mindset which accompanied the perceived obligation to the organization was more restricted than the moral duty of the *high affective-normative* profile. Employees within this profile noted that they owed it to the organization to work hard while at work, and occasionally work longer hours, but did not feel the same level of personal sacrifice that the *high affective-normative* profile employees did.

*“Stability, I like it that I have pretty much an 8–5 job, and that there is minimal overtime, I can leave work at work, I don’t have to bring it home and I’d like it if it stayed that way because to me, it gives to me a good work/life balance. And if I need time off, then I’ve earned that right to have that time off. While I am at work I give 100% but I don’t think I owe them anything anymore. I’m there by choice now, not obligation.”(Participant 8—state finance manager*

Interestingly, in the above comment the participant noted that her obligation has now been fulfilled and she is staying with the organization out of choice and not obligation. The nature of employee’s mindsets is consistent with value-based commitment which is in line with Meyer et al.’s [27] theorising. Employees who have strong affective commitment to the organization tend to experience greater autonomy in self- regulation; as a result, they are more likely to put their discretionary effort into work and remain with the organization [27].

The biggest difference between the moderate affective-continuance group and the higher affective-normative group was lower levels of value alignment and the absence of ideology-infused psychological contract. Comments indicative of the latter were only received from the high affective-normative participants. However, it is important to recall that while levels of affective and normative commitments among the moderate affective-continuance group were lower, these two components of commitment were not absent altogether. Rather, participants from the moderate affective-continuance group did still appear to have felt obligations, although these were not as strong or as broad in scope as those evident in the high affective-normative group. Thus, while comments from the moderate affective-continuance group could not be said to demonstrate a mindset of a moral imperative, they did reflect a positive sense of obligation to the organization, just not to the same degree as the high affective-normative group. For these reasons, it can be argued that the moral imperative versus indebted obligation dichotomy is too simplistic to capture the complexity of mindsets associated with various commitment profiles.

While moral imperative mindset apparent in the high affective-normative group relates strongly with an ideological value-infused psychological contract, the mindset of the moderate affective-continuance profile group aligns more closely with a relational psychological contract. In other words, although the high affective-normative and moderate affective-continuance profiles both have positive obligations associated with them, the scope and extent of these felt obligations differ between the two. The nature of mindset associated with obligation is closely tied to an employee’s psychological contract, which is in line with McInnies et al.’s [26] findings and Meyer and Parfyonova’s [2] theory concerning commitment profiles. When employees have an ideology-infused psychological contract, they are likely to have a high affective-normative profile and experience normative commitment as a moral imperative.

In contrast, when they have a relational psychological contract, they are likely to have a moderate affective-continuance profile (or one with moderate levels of affective and normative commitment and a low level of continuance commitment) and a mindset that reflects positive felt obligations but of a more restricted nature. In terms of a possible two faces of normative commitment, it appears that when employees have a relational psychological contract without high levels of value congruence, they are likely to have a moderate commitment profile but neither a moral imperative nor indebted obligation mindset. In addition to the absence of the strong value alignment of the high affective-normative profile, the moderate affective-continuance group made more comments emphasising other foci of commitment than the organization, such as co-workers, supervisor, and students or clients. One senior manager noted that his commitment was to his staff and not the organization:

“*The only obligations I have are to the people who work for me and those that I work with in my department–I don’t feel obligated to the larger organisation*. *I feel like we are a micro-cell of the organisation*. *I want to support my team–if I wanted more money or anything else I would leave and take up a mining job*. *The organisation itself is almost an irrelevancy–if my managerial team left to start our own business I’m sure that many of our staff would leave to join us*.*” (Participant 13- mechanical engineer)*

### Indebted obligation I: High continuance commitment

This profile group was expected to have a mindset characterised in terms of an exchange-based commitment [27]. An exchange-based mindset is focused on perceived cost, within the terms of the employment contract. It was apparent that the only employee with this profile engaged in behaviours that met only the minimum requirement to avoid costs. Despite the low level of normative commitment present in this profile, however, the participant’s comments clearly suggested a type of felt obligation, one with parallels to indebted obligation. This could be seen in this participant’s acknowledgement that the employer had provided the job, with reasonable pay, enabling the participant to do something he liked. Participant 4 indicated a definite reluctance to make any personal sacrifices for the organization. Nevertheless, the clear emphasis of this participant’s comments points to a mindset of exchange.

“*It is purely economic necessity*, *it is extremely difficult to find another job*, *so work for me is just a contractual thing–they pay me and I work for them*. *I mean I’m obligated in the sense that they have given me a job and they are paying me reasonably well and I like what I am doing and they make it possible for me to do what I am doing but I not going to stand in front of the firing squad for them*. *But there is a sense of obligation*, *a sense of well they have given me the opportunity to do what I like but then*…*” (Participant 4—Academic)*

### Indebted obligation II: Moderate continuance and normative

This profile group was expected to express the nature of their felt obligations as indebted obligation [2, 9]. However, while the moderate continuance-normative group had a greater intensity of felt obligation than the high continuance commitment group, as apparent from the higher level of normative commitment, the level of indebtedness felt was less clear. Obligations for participants in this group stemmed from the need to avoid the feelings of guilt, inconvenience, or loosing face [see 2, 9]. Additionally, this more “restricted” obligation was accompanied by less positive affect towards the organization and feelings that the organization and its agents did not necessarily recognise or value the employees’ contributions.

The pattern of responses was consistent with recent research findings [26] and theoretical development [2] on the association between OC and psychological contracts. Meyer and Parfyonova [2] proposed that the nature of obligation of employees with high continuance commitment would be more likely to reflect a purely transactional psychological contract. That is, employees with profiles featuring high continuance commitment were more likely to perceive their commitments to the organization as restricted to the explicit terms of their employment contract.

“*I don’t know if it’s because of a genuine recognition that what I do is good and valuable or if it’s simply given in these economic times*, *I’m simply easier and cheaper to keep on rather than go and recruit more people and because I can teach across so many different areas*. *(Participant 5 –system engineer)*

### Absence of obligations or uncommitted

The uncommitted profile reinforced the links between OC and psychological contracts. All four participants with an uncommitted profile identified a clear psychological contract violation as the cause of their low levels of commitment. A psychological contract violation refers to a situation where one party in a relationship perceives another to have failed to fulfil promised obligations [28, 29]. A senior human resource practitioner noted that she felt no obligation to her current organization because her promised role did not match the realities of her work:

“*None*! *I actually think they owe me*. *What I was told this role could be about is definitely not what it is*. *There has been a complete failure in the psychological contract–but of course they wouldn’t even know what that is*.*” (Participant 15 –human resource practitioner)*

Morrison and Robinson [19] argued that this definition focuses only on the rational, assessment of whether expectations have been met and fails to take into account the emotional aspect of expectation violation. They offered a distinction between the cognitive component and the emotional component, which they label as a psychological contract breach and a psychological contract violation respectively. Psychological contract violation is more personalised as rather than only failing to meet expectations, trust has been broken and promises have not been kept. A violation is an “emotional and affective state that may follow from the belief that one’s organization has failed to adequately maintain the psychological contract” [19].

### Multiple foci of commitment

All participants, irrespective of their commitment profiles, noted that they had commitments to a variety of foci within the organization, such as co-workers, supervisor(s), and students or clients. However, the qualitative data indicated that the participants’ commitment to these various foci differed by commitment profile. In comments from the high affective-normative profile group, commitment to the organization and other foci were given equal emphasis; in comments from the moderate affective-continuance group, the participants’ emphasis was on their commitment to foci other than the organization; in the high continuance commitment and moderate continuance-normative groups, there was no emphasis on any particular foci; and finally, in the uncommitted group there was an emphasis on absence of commitment to their organization.

## Discussion

The presence of commitment profiles offered a start in examining whether the dual normative commitment depending on relative level of affective and continuance commitments. The high affective/normative profile group described the participants’ relationship with, and felt obligations to, their organizations consistent with a moral imperative [2, 9]. Central to the mindset associated with this profile was the fulfilment of an ideology-infused psychological contract, high levels of perceived organizational support, and supportive leadership. Further, the high continuance commitment profile group described their relationship with their organizations consistent with an exchange-based mindset [27].

Previous research had only inferred employees’ mindsets from the pattern of survey results. The current study makes further substantial contributions through examining the mindsets associated with commitment profiles in which the levels of affective and continuance are not high. The moderate affective commitment profile participants described their relationship in terms of a relational psychological contract. Their perceived obligations to the organization were more restricted than the moral imperative felt by the high affective-normative group.

The biggest difference between the moderate affective commitment group and the high affective-normative group was lower levels of value alignment and the absence of ideology-infused psychological contract. Additionally, the moderate continuance-normative group reflected a purely transactional psychological contract, with employees restricting their organizational commitments to the explicit terms of their employment contract and engaging in behaviours that met only the minimum required to avoid costs and maintain face. The moderate continuance-normative group differed from the high continuance commitment group due to the presence of commitment in the moderate continuance-normative group and the absence of such commitment in the high continuance commitment group.

The uncommitted profile reinforced the links between OC and psychological contracts. All four participants with an uncommitted profile identified a clear psychological contract violation as the cause of their low levels of commitment. Findings related to moderate affective commitment, moderate continuance-normative, and uncommitted profile groups highlight the need for further theory developments on commitment profiles beyond the dichotomy of moral imperative and indebted obligation [3], and on the interaction of multiple foci [4].

Another strong theme in the current study was the complex manner, in which multiple commitment foci interact in all commitment profiles. Employees noted that they have commitment to a variety of foci within the organization, such as co-workers, supervisor and students/clients irrespective of their commitment profile. However, the alignment between commitment to these various foci differed by commitment profile. In the high affective-normative commitment profile the commitment to the organization and other foci was in alignment; in the moderate affective commitment group there was compatibility but the participants emphasis was on their commitment to foci other than the organization; in the high continuance commitment group there was no emphasis on any particular foci but no incompatibility either; and finally in the uncommitted group there was clear incongruence.

The complex manner in which multiple commitment foci interact is an area requiring further research. The future research can adopt the person-centred approach by answering research questions concerning how the components and various foci of commitment may combine, how employees of an organization may experience these components, and how groups characterised by various commitment profiles may exhibit differences in terms of other variables [4] and can further investigate the impact of when commitment to different foci are in conflict or are compatible with one another [4, 30]. Cooper-Hakim and Viswesvaran [30] noted that when there are multiple foci of commitment there is the potential for conflict between them. For example, Becker and Billings [21] discussed the potential for conflict (or compatibility) between commitment to local foci (supervisor and work group) and global foci (top management). Other examples of potential conflict between foci of commitment are between commitment to a profession or an organization [31, 32], and commitment to a union or an organization [33].

Conversely it has been argued that commitments to different foci can be compatible and even “mutually reinforcing under conditions where the goals and values of the foci are overlapping” [4]. In cases where fostering a moral commitment to an organization is not possible, it may be possible to build a strong moral commitment to other foci (e.g., students, profession, co-workers) whose goals overlap with those of the organization [see 34].

### Limitations and future research

Qualitative research is often criticised for lack of generalizability due to small sample sizes and non-representative samples [35]. However, the main purpose of the investigation was to clarify the nature of the mindsets associated with employees’ commitment profiles, where mindsets are derived from the perception of obligation to an organization rather than by generalising from the sample to the population [35, 36].

In addition, this study attempted to reduce potential subjectivity issues by using a semi-structured interview protocol. Open-ended questions were formulated and the responses made by participants were recorded. These responses prompted additional questions that sought to illuminate or to enlarge on points raised by the participant. Objectivity was subsequently assessed in the analysis stage by calculating interrater agreement.

One of the key strengths of the current study was the application of qualitative research methods to investigate OC. The qualitative differences among the commitment profiles indicated that the interaction of the commitment components may be more complex than the dual nature of normative commitment proposition suggests. Hence, it is necessary that future research on OC utilise a mixed- methods approach to reveal more in-depth understanding of commitment profiles and context effects. In accordance with the present findings, future research on commitment profiles could utilise longitudinal research designs to investigate changes in employees’ commitment profiles, associated mindsets, psychological contracts, and obligations over time.

## Conclusion

The qualitative differences in employee mindsets associated with commitment profiles suggest that the interaction of the commitment components is more complex than the dual nature of normative commitment propositions suggests. In summary, this study argued that to further our understanding of commitment profiles the nature of employee mindsets associated with different commitment profiles needed to be qualitatively examined. Do employees with different commitment profiles describe their “mindsets” differently as predicted by commitment profile research? Simply put, yes they do but in a more complex manner than previously theorised.

## Supporting information

S1 Appendix (DOCX)
