# Supplementary material for: Qualitative differences in the mindsets associated with dual nature of normative commitment
Source: PLoS One. 2021 Jun 17;16(6):e0251193. doi: 10.1371/journal.pone.0251193 (PMC8211279; doi:10.1371/journal.pone.0251193)
Supplement: S1 Appendix — (DOCX) [file pone.0251193.s001.docx]

**S1 Appendix**

Interview protocol

*Employment Relationship/Psychological Contract*

1. What are your feelings toward your organization?
2. What do you like most about working here?
3. What do you like least about your working here?
4. What do you think your organization expects of you?

Prompts: A level of technical/generic skills/Work output – broad/narrow?/working hard /Certain number of years tenure

1. What did you expect to receive from your employer, e.g. support, high pay, etc? *(prompts available along relational, balanced, transactional contract types)*

Prompts: Training/development/career development/Type of work – broad/narrow?/Type of supervision /A supportive environment – e.g. understanding, can ask questions freely, constructive feedback provided/recognition /Certain pay and conditions /Stability of employment

1. Overall, have your expectations been met/exceeded/not met/a bit of all over time?
2. How does this make you feel?

*Obligations*

1. What obligations do you feel towards your employer?
2. What creates/influences this sense of obligation?
3. How do these obligations make you feel?
4. How do you fulfil this obligation/what do you do to meet this obligation?
5. What motivates you to meet/fulfil these obligations?
6. Is there anything that would change your obligations to your organization
7. In summary, would you describe your obligation as either:

- A moral duty/imperative (e.g., a strong sense or desire to pursue a course of action to benefit the organization or that is the right thing to do) or more as
- A sense of indebtedness (indebted obligation or something that must be done to meet obligations and/or save face)?

*Supervisor/Leadership*

1. Tell me about your relationship with your supervisor

Prompts: Ask about: time spent with them/ direction given/ feedback/ the type/complexity of work given/ conflict/ autonomy/ approachability/friendliness/general support, etc.

1. How would you describe your supervisor’s leadership style? (Prompts available along transactional and transformational lines)
2. How important was your supervisor/s in shaping your expectations/perception of organization’s obligations?

*Motivation/Forms of Regulation*

1. What is your primary motivation to meet work goals?

Prompts:

• External regulation - to attain externally controlled rewards or avoid punishment

• Introjected regulation – to meet one’s own or other expectation or to avoid shame

• Autonomous regulation – to achieve valued goals, self-expression, meaningful/relevant outcomes

1. Is there anything that you want to add or stress that might help me understand your relationship and feelings towards your employer?
